# Endurance of Damping Properties of Foam-Filled Tubes

**DOI:** 10.3390/ma8074061

**Published:** 2015-07-07

**Authors:** Matteo Strano, Alessandro Marra, Valerio Mussi, Massimo Goletti, Philippe Bocher

**Affiliations:** 1 Politecnico di Milano, Dipartimento di Meccanica, via La Masa 1, Milan 20156, Italy; E-Mail: alessandro.marra@mail.polimi.it; 2 MUSP Lab, via Tirotti 9, Piacenza 29122, Italy; E-Mails: valerio.mussi@musp.it (V.M.); massimo.goletti@musp.it (M.G.); 3 École de Technologie Supérieure, Département de génie mécanique, Montréal H3C 1K3, Canada; E-Mail: Philippe.Bocher@etsmtl.ca

**Keywords:** cellular metals, advanced pore morphology, vibrations, cyclic bending, interaction effect, metal foam

## Abstract

The favorable energy-absorption properties of metal foams have been frequently proposed for damping or anti-crash applications. The aim of this paper is to investigate the endurance of these properties for composite structures, made by a metal or a hybrid metal-polymeric foam used as the core filling of a tubular metal case. The results of experimental tests are shown, run with two types of structures: 1) square steel tubes filled with aluminum or with hybrid aluminum-polymer foams; 2) round titanium tubes filled with aluminum foams. The paper shows that the damping properties of a foam-filled tube change (improve) with the number of cycles, while all other dynamic properties are nearly constant. This result is very important for several potential applications where damping is crucial, e.g., for machine tools.

## 1. Introduction

The use of parts made of an outer metal case (e.g., a steel tube) and a core filled with metal (e.g., aluminum) foam was frequently proposed in the technical and scientific literature for several applications. If the metal foam has a closed cell morphology [1], the part can be used especially for lightweight structural applications. Aluminum foams are very interesting as a filler because they are lightweight [2], less expensive than honeycomb structures [3] and they show higher strength and toughness than polymeric foams. Hybrid aluminum-epoxy foams made by the Hybrid Advanced Pore Morphology (APM) process developed at Fraunhofer IFAM in Bremen and described in [4,5] are a composite combination of spheroidal aluminum foam pellets, bonded by an epoxy expandable adhesive. They are conveniently used as fillers of large metal structures, because the final foaming operation can be performed at low temperature, reducing potential thermal distortion and softening of the outer case structure [6].

One of the most promising applications of closed-cell metal foam-filled structures is in anti-vibration or damping devices, e.g., for machine tools [7] or (potentially) helicopters. The application deals with the peculiar ability of foam-filled structures in absorbing and dissipating mechanical energy. Protection devices benefit from the ability of foam-filled structures to absorb and dissipate mechanical energy in the large strain regime [8], if loaded in compression, bending and torsion (not in tension). Damping devices benefit from the property of the metal foams of easily dissipating mechanical energy at small strains [9], under all stress states. In vibrational loading with low amplitude, the mechanical energy is dissipated because of a hysteresis cycle of the material, which in cellular metallic materials is enhanced by localized stresses at the cell walls. 

### 1.1. Stability of Damping Properties of Cellular Metals

Similar to solid metals, metal foams may fail by fatigue, depending on the level and state of stress and as a function of the number of cycles. Different authors, e.g., [10], have studied the fatigue limits of Al-Mg-Si foams produced by the powder compact melting technique. Zettl *et al*. [11] have found that fatigue cracks initiate in the interior closed-cell structure at holes or pre-existing cracks in the cell walls or in areas where the cell walls are thin, and the surface layer subsequently cracks. At the endurance limit, fatigue cracks may initiate in cell walls, however, they are trapped at nodes of cells. If loaded below the endurance limit, expressed in terms of strain amplitude, Al-Mg-Si foams may survive for 10^9^ cycles or more. Failure by fatigue is the ultimate catastrophic event that follows a number of damage phenomena that start occurring inside the material at the microscopic level, already at low strain and low cycle number. For this reason, well before their failure by fatigue, metal foams might change their mechanical properties during their working life. Damping is a result of the same damage phenomena (small-scale plastic deformation, microscopic cracking, internal friction, thermo-elastic effect), that microscopically change the morphology of the foam and may, in the long run, lead to failure by fatigue [12]. As damage is an accumulative process, it is interesting to investigate the stability of the material as it is loaded cyclically, *i.e.*, if and how the damping properties evolve for cellular materials. Few researchers in the past have studied the stability (or constancy) of damping properties of metal foams. Golovin *et al*. showed that the instability of damping is frequently observed in cellular metals even for relatively small strain amplitudes [13]. Increasing the number of vibrations has a positive effect as it leads to an increase of dissipated energy. Dattoma and co-workers used the increasing damping ability of metal foams with a number of cycles in order to develop a method for predicting the residual fatigue life of pure aluminum foam samples [14,15]. They showed that the natural frequencies of metal foams tend to decrease with the number of loading cycles.

### 1.2. Stability of Damping Properties of Foam-Filled Structures

In damping applications, the intrinsic properties of cellular metals in general and aluminum foams especially are dramatically enhanced when the cellular material is not used alone, but used as a filler of restraining outer cases, such as metal tubes or sandwich skin panels. The purpose of the outer case is to provide strength and stiffness to the structure; the purpose of the foam core is to provide energy-absorption properties, according to the mechanism described above. Tubular structures are particularly well suited because of their closed cross-section. At large strains, both in axial and lateral impacts, aluminum foam-filled tubular structures show a higher collapse load and a higher energy absorption than those of the tube and of the foam filling considered individually. This behavior, often called “interaction effect,” has already been frequently demonstrated and described (e.g., in [16] or [17]). Conversely, the literature on the behavior of small strains of constrained cellular metals is very limited, although there are hints that the endurance and the damping properties of metal foams are positively influenced by the presence of an outer case. As an example, according to Kolluri *et al.* [18], the compression–compression fatigue behavior of closed-cell aluminum foam with lateral constraint reduces the effective maximum stress experienced by the foam specimen during fatigue loading. Their study suggests that the damage that accumulates during fatigue does not affect the energy-absorbing ability of a structure. Harte *et al*. [19] studied the fatigue strength in four-point bending of sandwich beams with an aluminum alloy foam core. They found that a reduction in the strength of sandwich beams exists for cyclic loading compared to monotonic loading, but the set of possible collapse mechanisms does not change.

To the authors’ knowledge, no literature is currently available on the stability of damping properties of structures where the foam is used as a filler, which is indeed a very important case from an application point of view. It is useful to consider that, if an aluminum foam is used as the core bar of a metal tube of higher strength (e.g., steel or titanium), the greatest portion of the load in bending is taken by the outer case. In this configuration, the foam does not have any significant load-bearing purpose in regular use, and it only serves as a damper and as a safety material in case of accidents. However, even if the structure can be easily designed to work below its safe-life fatigue limit strain, its energy-absorption properties can still be altered during its lifetime. If the foam filler is not bonded to the outer case, energy is dissipated at the interface by frictional work. This frictional work at the interface might be an additional and useful damping mechanism that is obviously not present if the foam is considered alone. In bending modes, the surface in contact with the tube is also the most stressed region of the foam, therefore any microscopic modification that may induce changes in damping properties would necessarily start from the interface surface.

The first purpose of this paper is to investigate the stability of the energy-absorption properties of tubes filled with different kinds of foams, subject to loading at small strains during the life of the components. Indeed, this is to simulate the continuous exposure to vibrations, e.g., produced by the rotation of a milling spindle or by the rotation of a helicopter rotor, and verify whether these useful properties of foam-filled structures deteriorate, remain constant or improve during their lifespan. In doing this, steel tubes with square cross-sections filled with aluminum and hybrid aluminum-polymer foam (Section 2) were tested. The second purpose is to study the role of the interface condition between tube and foam filling in determining the damping properties and the cyclic loading response of these structures. To this aim, smaller titanium tubes with round cross-sections, filled with aluminum foam, were tested (Section 3).

## 2. Damping Stability of Foam-Filled Tubes

**Figure 1:**
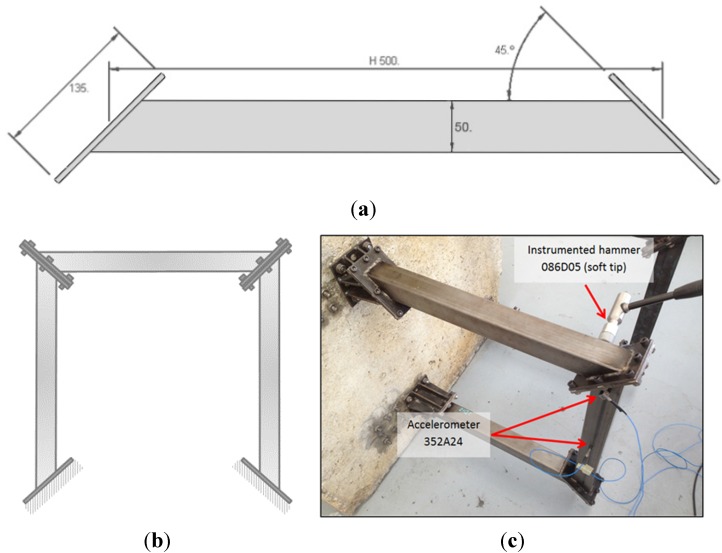
(**a**) Dimensions of steel tube; (**b**) Schematic representation of the model of portal composed by three samples; (**c**) Experimental setup for the frequency response functions (FRF) estimation along with accelerometer and impact point positions.

A testing structure is used for testing the damping properties of foam-filled tubes, designed in a previous work [6]. The structure is made of three square cross-section steel tubes filled with aluminum foam, assembled as in Figure 1.

This structure is fixed to a heavy mass (about 2300 kg) concrete block, with the purpose of simulating the behavior of a portal or gantry of a machine tool. Initially, the as-built structure was tested with an impact hammer in order to find the modal parameters. Then, each of the three tubes underwent cyclic bending tests, in order to simulate a portion of the life of this component at work. Finally, the structure was assembled again and a new modal analysis was performed in order to understand the evolution of modal parameters, especially the damping ratio.

### 2.1. Production and Properties of the Foam-Filled Tubes

The geometrical and material properties of the members of the tested structure are given in Table 1. Before filling the structures with the APM foam, each tube was cleaned with alcohol and then dried. The tube was then filled with aluminum foam spheres coated with a thermally activated adhesive which contains a chemical foaming agent produced by Fraunhofer IFAM (Bremen, Germany) using materials and a process described in [4]. The filled tubes were placed inside a furnace preheated at 160 °C and kept at this temperature for 3 h in order to foam and cure the epoxy adhesive. Then the tubes were removed from the furnace and allowed to cool in air. Two different final densities of the hybrid foams were obtained using APM precursor materials with a nominal bulk density of the coated metal foam spheres of 470 kg/m^3^ and 600 kg/m^3^ respectively. The nominal diameter of the aluminum foam spheres was 6–7 mm diameter and foam composition was AlSi10.

The steel tubes filled with the pure aluminum foam were left untreated. Foaminal AlSi10 commercial precursor was placed horizontally inside the tube. The tubes were foamed in a furnace preheated at 700 °C for about 12 min and then cooled in a compressed air flux.

**Table 1:** Properties of foam-filled steel members.

Materials		Properties
Outer case: steel tubular elements		Yield stress 250 MPa, length 500 mm, square cross-section 50 mm × 50 mm, 2 mm wall thickness
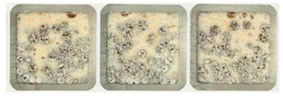	Filling: APM L	Low-density hybrid foam (estimated ρ ~ 490 kg/m^3^), made by hybrid aluminum-epoxy foam
	Filling: APM H	High-density hybrid foam (estimated ρ ~ 590 kg/m^3^), made by hybrid aluminum-epoxy foam
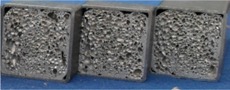	Filling: AlSi10	Alulight Foaminal AlSi10 foam (estimated ρ ~ 550 kg/m^3^)

### 2.2. Cyclic Three-Point Bending Tests

**Figure 2:**
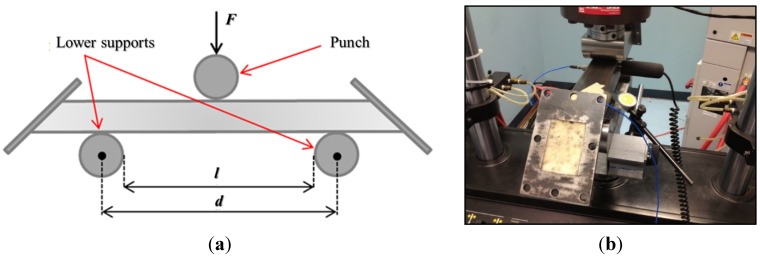
(**a**) Schematic representation of the three-point bending test (*l* = 216 mm, *d* = 271 mm); (**b**) Position of the ultrasonic detector.

Cyclic three-point bending tests were executed on the foam-filled steel samples in order to simulate a number of work cycles. The machine used for the cyclic bending is an MTS 810 (MTS Systems Corp., Eden Prairie, MN, USA), which is controlled in force. The test setup is shown in Figure 2a. An ultrasonic detector UE Systems UltraProbe 10000 was installed during the tests for monitoring the acoustic emissions of the structure, in order to detect the potential forming of macroscopic cracks inside the cellular material. The signals were acquired in a range between 27.5 and 32.5 kHz and were converted in the field of audible frequencies. The system was installed as represented in Figure 2b using a magnetic attachment that permits consistent results by eliminating variables such as angle of approach and contact probe pressure. The ultrasound detector was connected with an analog digital converter (THOR Analyzer PRO: DT9837-13310, Hyatt Industries Ltd., Vancouver, BC, Canada).

#### 2.2.1. Preliminary Tests

**Figure 3:**
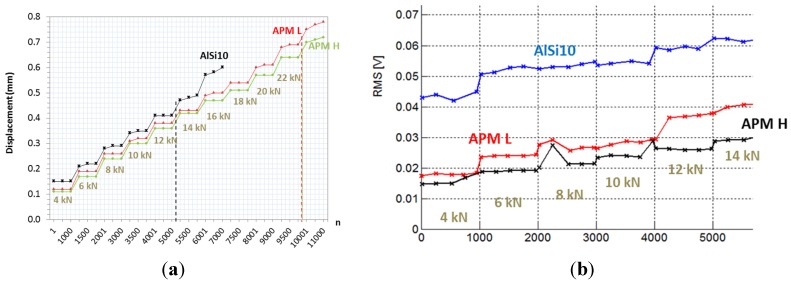
(**a**) Displacement *vs.* cycle number with staircase loading of foam-filled square tubes; (**b**) RMS of the acoustic emission *vs.* cycle number with staircase loading of foam-filled square tubes.

Preliminary samples were tested in order to determine the maximum load that the structure can bear without damage or plasticization. A staircase-loading approach was used, increasing the load by 2 kN every 1000 cycles, starting from an initial load of 4 kN. In Figure 3a, these preliminary results are reported. The figure shows that, up to 12 kN, the displacement of the tube is constant at each step of the staircase. After 12 kN, the tube filled with only aluminum foam becomes unstable. After 22 kN, the displacement of the other two structures starts to increase within a single loading step. The tube filled with AlSi10 foam is weaker, supposedly because the outer steel case was softened by the foaming cycle at 700 °C for 12 min, while the other steel tubes were only heated at 160 °C during the production phase. The lowest displacement is exhibited by the higher density APM foam-filled column. The RMS (Root Mean Square) of the acoustic emission, converted into the range 1–5000 Hz, continuously and slowly increases with the increase of displacements. The signal presents some small and occasional spikes, in proximity of the load jumps (Figure 3b). The signal does not show indications of macroscopic damages or cracks inside the foam.

#### 2.2.2. Final Tests

The gantry shown in Figure 1 is made of three parts; since three types of foam filling are available, a total of nine structures were tested. The tubes underwent 10,000 bending cycles each, with a minimum load of 2 kN, a maximum load of 10 kN, a sinusoidal load profile and a loading cycle frequency of 1 Hz. The value of 10 kN was selected, thanks to preliminary tests, in order for no macroscopic damage to occur on the tubes during the tests. As expected, the displacement of the punch after the first and the 10,000th cycle remained unchanged for each tube, with an average of 0.415 mm for the AlSi10-filled samples and 0.333 mm for the APM L and APM H samples.

During the final tests, the RMS signal remained practically constant, with no jumps, indicating that no relevant damage occurred inside the materials. Although the study was conducted only with respect to a low cycle behavior (10,000 cycles), there seems to be a lower loading limit, below which no detrimental effects are evident on the endurance of foam-filled structures. This behavior is in line with previous research works in the case of samples made entirely by metal foams [11].

### 2.3. Modal Analysis

The setup shown in Figure 1 was used for the determination of vibration modes, before and after the cyclic tests. The low-frequency modes were tested with a soft tip mounted on the instrumented hammer described in Section 2.1. The middle and high (membrane) frequency modes were measured with a lighter hammer with harder tip. The modal analysis performed was carried out with PCB^®^ instrumented hammers and accelerometers (086C04, 086D05, 352C23, 352A24) and National Instruments^®^ Data Acquisition Device (2 × 9234 with cDAQ 9178). The frequency response functions (FRF) were computed with the commonly used H1 estimator [20].

**Figure 4:**
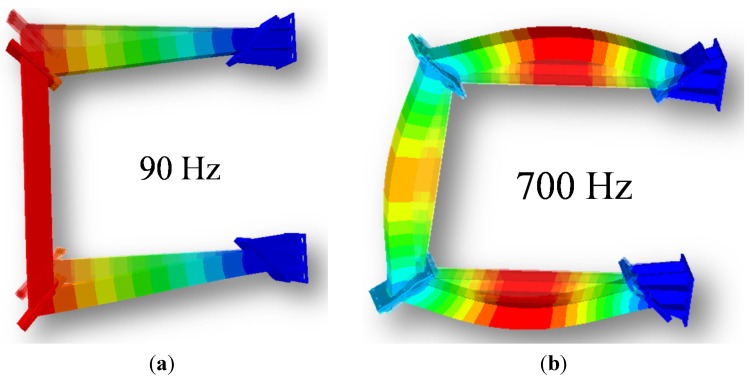
Dimensionless displacement vectors (*i.e.*, eigenvectors) for the selected low-frequency (**a**) and high-frequency (**b**) modes of the gantry structure.

The modal parameter identification was carried out by means of the manual selection of the peaks of interest according to the algorithms described in [20]. The identification was carried out one peak at a time in a recursive way to separate the various contributions. Only two modes were selected, one in the low-frequency range (labeled 90 Hz) and the second one in the high-frequency range (labeled 700 Hz). It is worth noting that the high-frequency mode is a parabola starting and ending at near-zero deformation, with relevant bending of the structure (Figure 4). On the contrary, the low-frequency mode is a straight line from the constraint to the free end. High-frequency modes (membrane modes) are not present, *i.e.*, they are suppressed by the foam filling (while they are present for the empty tube).

The considered performance indicators are the typical modal parameters: mode frequency, dynamic stiffness, modal mass and damping ratio (defined as the ratio between the damping coefficient c and the critical damping coefficient).

(1) $$DR=\frac{c}{2\sqrt{k\cdot m}}$$

**Figure 5:**
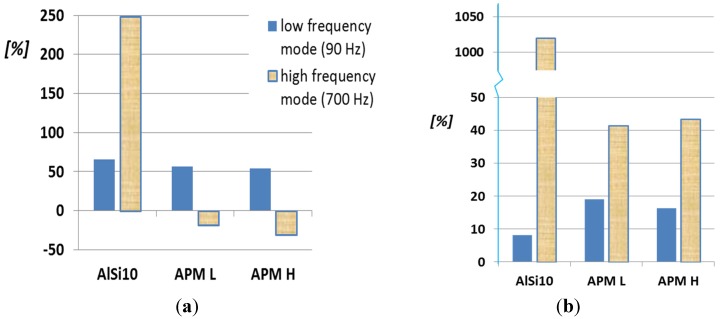
(**a**) Percentage variation of damping ratio DR of filled structures, with respect of empty tube [6]; (**b**) Percentage variation of DR of foam-filled structures after 10,000 bending cycles, with respect to hollow tubes.

The values of the modal parameters measured before and after the 10,000 bending cycles were compared. The only parameter that has changed is the damping ratio. It is interesting to observe that the damping ratio increases for all structures after all bending tests. In other words, despite no macroscopic damage being detected and despite all other modal parameters remaining unaltered, the damping ability of foam-filled structures improves with an increasing number of cycles, as shown in Figure 5b. At low frequency, the increase in damping ability is similar for all structures: the cyclically bent tubes outperform the hollow tubes ranging from an increase in damping ratio of +8% up to +19%. For the high-frequency mode, the APM-filled tubes after bending tests are significantly better than the hollow ones (about +42%), but the most surprising result is due to AlSi10 foam-filled structure. The increase in damping ratio is measured as +1020%, *i.e*., the damping ratio is ten times larger for the cyclically bent tube than for the hollow tube. The most probable cause of this greatly improved damping is due to frictional behavior at the interface between the filling and the outer case. In the case of the AlSi10 foam, while cyclic bending tests are run, the most stressed regions of the structure are the intrados and the extrados of the foam bar, *i.e.*, the top and bottom flat interface surfaces with the outer tube. It is plausible that the continuous sliding flattens microscopic irregularities at the interface, decreasing the coefficient of friction and further facilitating relative movement. The reduction of the static coefficient of friction enables greater relative movements at the interface under the bending stress, which dissipates more energy by frictional work. Conversely, the hybrid APM fillers are bonded (glued) to the skin, and this bond is not disrupted by the cyclic test; hence, no work can be dissipated by friction. At low frequency, where no bending takes place (as shown in Figure 4), the APM foams outperforms the aluminum foam.

The dynamic analysis shows how the damping capacity not only does not worsen, but also increases significantly after the structure undergoes several work cycles. This result is somehow in agreement with the findings in references [11,12,13]. However, the previous works were limited to samples made of metal foam only, hence no interface or interaction effects between foam core and metal skin could be observed. When cellular metals are tested alone, shifts can be observed of all modal parameters. On the contrary, the present study indicates that in foam-filled structures, the mode frequency, the dynamic stiffness and the modal mass are virtually unchanged before and after the endurance tests, and only the damping ratio significantly increases.

## 3. The Interface Effect

The improvement of damping properties of metal foam-filled structures is presumably due to two separate effects: the hysteresis on the foam filling and the frictional work at the tube-foam interface. It is therefore interesting to understand how influent the frictional effect can be in determining both the damping ability and the mechanical response to fatigue of filled tubes. For this reason, tubes were produced with two different interface conditions between the surfaces of foam and tube: a free or “as-produced” interface resulting from the foaming process, and a bonded interface using an epoxy glue. The use of a bonding agent was used to cut off the effect of friction on damping.

### 3.1. Test Materials

The foam-filled steel tubes studied previously could not be used for this study. Indeed, both the square geometry of the section and the intermetallic layer between aluminum foam and steel prevented a proper bonding of the two surfaces. For this reason, a different configuration was used for the structures, using round titanium tubes as the outer case. As explained by Yan *et al*. [21], there is a low solubility at the interface of solid titanium and molten aluminum, thus no intermetallic is observed after the foaming process. In this configuration, the foam filling can be easily extracted from the tube with no damage, allowing for the manual bonding. The tubes are made by titanium grade 2, outer diameter 40 mm, wall thickness 1.2 mm. The foaming was performed with Alulight Foaminal commercial precursors, placed horizontally inside the tubes, foamed in an air convection furnace preheated at 750 *°*C for about 13 min and finally cooled down in a compressed air flow. Tubes with two different values of density were produced: samples labelled AlSi10 L with a nominal density of 560 kg/m^3^ and samples labelled AlSi10 H with a nominal density of 815 kg/m^3^. The labels L and H indicate respectively low and high density. The final length of the filled tubes is 213 mm. As stated above, some of the tubes have been bonded to the aluminum foam by gluing: first the foam was extracted from the tube, then the titanium and foam surfaces were brushed and cleaned with alcohol and acetone. Finally, the adhesive Hysol^®^ 9466 was applied and the foam was inserted into the tube. After 48 h of glue polymerization, the bonded tubes were ready to be tested.

### 3.2. The Influence on Damping

#### 3.2.1. Methods

Both experiments and numerical (finite element model, FEM) simulations were conducted in order to investigate the damping characteristics of the tubes, tested in a free-free boundary condition, in order to avoid adding any outer source of damping.

**Figure 6:**
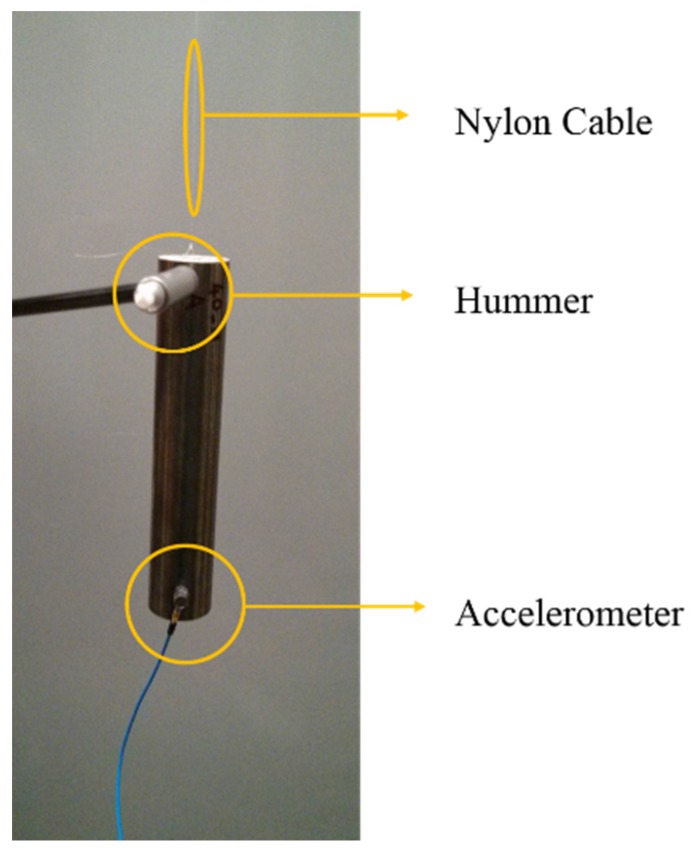
Experimental setup for free-free tests.

Experimentally, each specimen was connected to one extremity of a nylon cable while the other extremity was connected to a cantilever so that the tube was suspended in the air. A high sensitivity (100.8 mV/g) mono-directional accelerometer was attached on the bottom side of the tube while the point of excitation for the hammer was on the top side as in Figure 6. The hammer was equipped with a hard spherical steel tip in order to excite frequencies up to 10 kHz. Each specimen was hammer tested five times and the responses were averaged, calculating the final FRF. Three specimens were tested for each combination of the two factors density (low and high) of the foam and interface condition (natural or bonded), for the 12 specimens tested.

An FEM model was built with the software Abaqus, in order to have an idea of the natural frequencies and the shape of the related transverse modes of vibration of these tubes. In this analysis, a tied constraint between the tube and the foam was set; this condition well simulates the bonded interface but is quite far from the naturally foamed specimen. Indeed, the aim of this simulation was not to obtain a precise prediction of the modal parameters, but only an approximate indication of the natural frequencies and modes shape. The 3D shell homogeneous elements were used for the tube, wherease the 3D solid homogeneous ones were used for the foam. For both types of element, an average side length of 3.5 mm was used. An elastic material model was associated to both materials; the low-density foam was given an elastic modulus of 4000 MPa, the high-density foam was given a modulus of 6400 MPa, known from previous studies.

#### 3.2.2. Results

**Figure 7:**
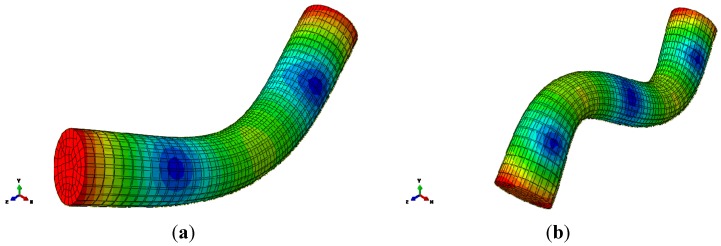
Shape of vibration modes (dimensionless displacement vectors) for a glued interface tube filled with a high-density foam; (**a**) first mode; (**b**) second mode.

According to the results of numerical simulations, the shape of the first two vibrational modes is given in Figure 7 while the related values of natural frequency are about 3640 Hz and 8300 Hz for the tube filled with low-density foam and about 3470 Hz and 8020 Hz for the high-density sample.

This small difference between low and high-density filling is due to a general increase in mass not accompanied by an equivalent increase in stiffness, thus making the natural frequencies decrease. In [22], Ashby proposes a quadratic scaling law between the young moduli of a metal foam beam and its relative density (see Equation (2)). The frequencies of flexural vibrations of a beam follow Equation (3), where *C*_1_ is a constant depending on the boundary conditions, *I* is the momentum of inertia of the cross-section, *A* is the area of the section, *l* is the length of the beam, *E* is the Young modulus and ρ is the density of the foam. Since the Young modulus is proportional to the second power of the density, the natural frequencies of a foam beam will be proportional to ρ^1/2^.

(2) $$E=\left(0.1÷1.0\right)E_{\text{s}}\left(0.5{\left(\frac{\text{ρ}}{{\text{ρ}}_{\text{s}}}\right)}^{2}+0.3\left(\frac{\text{ρ}}{{\text{ρ}}_{\text{s}}}\right)\right)$$

(3) $$f_{1}=\frac{C_{1}}{2\pi }\frac{1}{l^{2}}\sqrt{\frac{EI}{A\text{ρ}}}$$

Nevertheless, the tubes studied in this work have a flexural stiffness (*EI*)_tube_ that is the sum of the one related to the external titanium tube (*EI*)_Ti_skin_ and the one of the foam filling (*EI*)_foam_, considering a perfect coupling between the internal surfaces.

(4) $${\left(EI\right)}_{tube}={\left(EI\right)}_{Ti\_skin}+{\left(EI\right)}_{foam}$$

**Figure 8:**
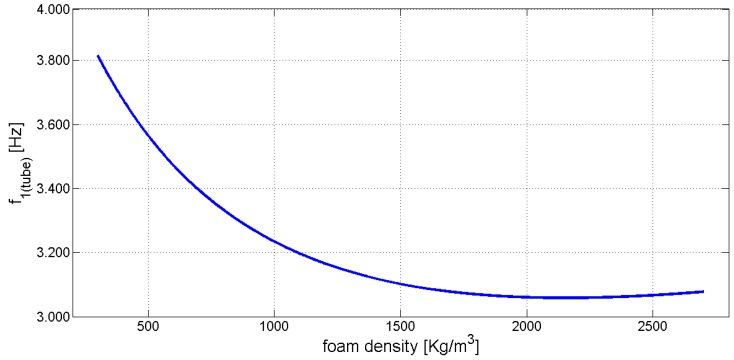
Value of the first resonance frequency of filled tubes scaling as a function of the density of the foam filling (analytical law): 2 mm of titanium external skin and 38 mm of entire tube diameter.

Being the young modulus of titanium and aluminum metal foam, respectively 105,000 MPa and 4000 or 6400 MPa (lower or higher-]density foam), the titanium external tube gives the prevalent contribution to the total flexural stiffness of the structure. Indeed, in accordance with Equations (2)–(4) and considering the geometrical parameters of the tubes, the value of the first flexural natural frequency of the structure can be plotted as a function of the density of the foam filling (Figure 8).

**Figure 9:**
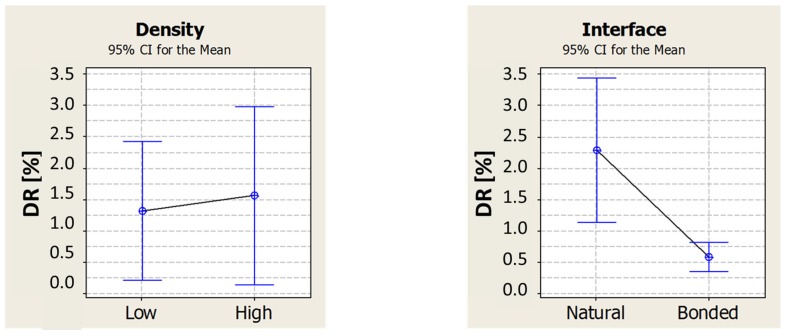
Effect of foam density and interface condition on damping ratio in the low-frequency range (1st mode of vibration).

**Figure 10:**
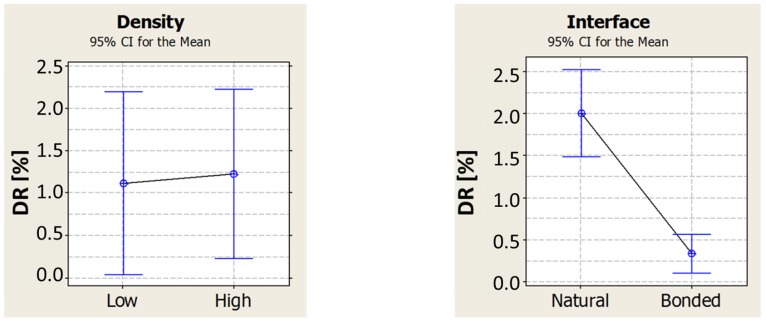
Effect of foam density and interface condition on damping ratio in the high-frequency range (2nd mode of vibration).

The simulation information helped to set the testing parameters and to analyze the response properly. The parameter considered for estimating the damping is again the damping ratio DR. The procedure and methods used for computing the FRF, natural frequencies and damping ratio are the same as the ones described in Section 2.3. A sensible difference can be immediately appreciated just by looking at the shape of the FRF functions. Indeed, the glued tubes show a clean response with easy recognizable resonance frequencies, close to the simulation results; the natural interface tubes have a noisier response, showing many other peaks. This is probably due to friction between the external foam surface and internal tube one, which introduces noise. Moreover, the detachment between the two surfaces triggers many other resonance frequencies related to membrane modes of vibration. Due to this unpredictable response, it was decided to define an interval around the simulation value of natural frequency, and investigate the damping of the most evident resonance frequency in the interval. The first interval was set between 2500 and 4000 Hz while the second one was between 6000 and 8500 Hz. A value of the damping ratio was computed for each of the twelve specimens and in each interval. In analyzing the results, the focus is on investigating the effect of the density of the foam filling and the interface condition. This was done for the first (low resonance) and the second (high resonance) frequency interval separately. Figure 9 and Figure 10, respectively for damping in low and high-frequency ranges, show how the change in foam density seems to have no effect, whereas the interface condition is strongly changing the damping ratio of these tubes. A statistical analysis of the data (ANOVA) confirms that, for a confidence level of 95%, the density has no effect on damping while the interface condition has a significant effect.

### 3.3. Cyclic Three-Point Bending Tests

Cyclic three-point bending tests were executed on the foam-filled titanium samples, in order to simulate a certain number of work cycles. The machine and setup used for the cyclic bending are the same as described in Section 2.2, with *l* = 155 mm and *d* = 210 mm.

**Figure 11:**
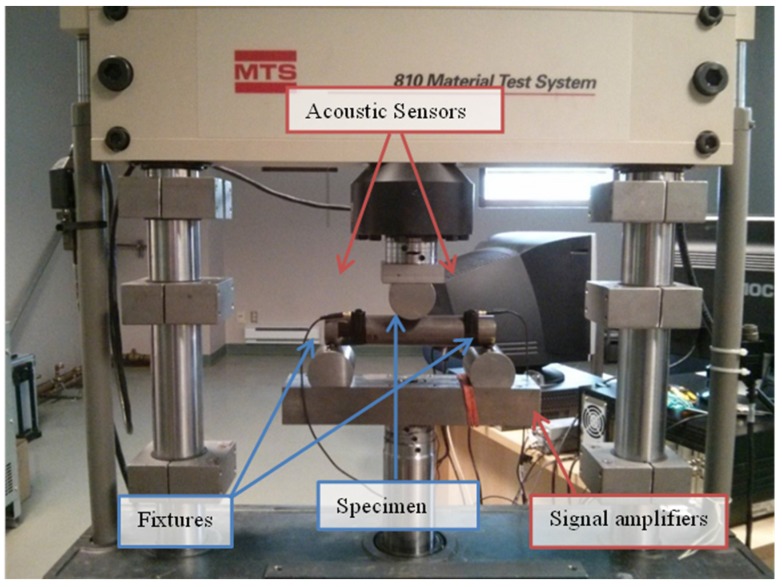
Setup for cyclic bending at constant load test on cylindrical foam-filled tubes.

During the tests, the acoustic signal was recorded, hoping to get some information about the microstructural failures and the overall energy absorbed during the cyclic loading. Acoustic emissions measurement were carried out using devices provided by Physical Acoustic Corporation (PAC). Two sensors (model Micro-80 PAC, bandwidth 100–1000 kHz) acquired the acoustic signal. Before each test, the quality of the coupling was verified through a Nielsen-Hsu pencil lead break. These sensors were taped to the tube approximately 35 mm away from the supports but on the upper side of it. A silicon adhesive gel was employed as a coupling agent between the surface of the sensor and the tube in order to improve the quality of the signal and to avoid friction between the two surfaces. The signal of each sensor was then sent to an amplifier that gave 40 dB of gain to the voltage signal, and then to the PCI card, able to receive two signals in input. The PCI card’s sample rate was set to 2 MHz, while the acoustic threshold to filter background noise was set at 40 dB. The quality of the measured AE data depends mainly on the choice of the waveform timing parameters, namely the peak definition time (PDT), the hit definition time (HDT) and the hit lockout time (HLT) [23]. The values of the timing parameters employed are PDT = 300 μs, HDT = 600 μs and HLT = 1000 μs, in accordance with the manual specifications for detecting cracks in metallic materials. The setup is visible in Figure 11.

#### 3.3.1. Step Loading Preliminary Tests

**Figure 12:**
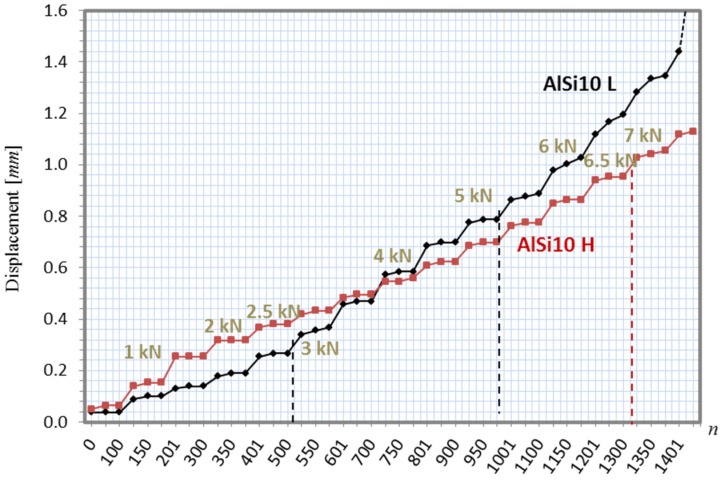
Displacement *vs.* cycle number with staircase loading of foam-filled tubes.

Foam-filled tubes with natural interface (AlSi10 L and AlSi10 H respectively for low and high density of the foam filling) were preliminarily tested in order to determine the maximum load that the piece can bear without damage or plasticization. A staircase-loading approach has been used, increasing the load by 0.5 kN every 100 cycles, starting from an initial load of 0.5 kN, with a sinusoidal loading pattern at a frequency of 1 Hz. The results in Figure 12 show that the tube filled with the lower density aluminum foam becomes unstable at 3 kN, *i.e*., the displacement increases at constant load. At 6.5 kN, the heavier structure becomes unstable as well. In conclusion, for both types of structures, there is an upper limit (2.5 kN), below which the displacement is nearly constant with constant load. No macroscopic damage should be expected below this limit. Consequently, constant loading tests were planned with the safe value of 2 kN, as described in the following subsection.

#### 3.3.2. Constant Loading Test

Cyclic bending was performed with a maximum load of 2 kN for 50,000 cycles, with a preload of 0.5 kN, a sinusoidal load profile and a loading cycle frequency of 10 Hz. Four specimens were tested, one for each combination of interface condition and density of the foam filling, namely AlSi Ln (low density, natural interface), Alsi Hn (high density, natural interface), AlSi Lb (low density, bonded interface) and AlSi (high density, bonded interface). Table 2 shows the results in terms of displacement for each tested specimen: *d*_max_ is the displacement at the maximum load in each cycle. It is a result of the overall stiffness of the structure. The results show that higher density samples are stiffer (as it was obvious) and that structures with a glued interface are stiffer than structures with a frictional interface, where relative motion is possible between the foam and the tube.

Moving from the first to the last cycle, the *d*_max_ values mildly increase for each tested structure. *∆d*_max%_ is the percentage increase of maximum displacement recorded between the first and the last cycles of the test. The results show that, at least in percentage terms, the stiffness of structures with a lower density is more stable over the testing time, while there is no clear difference between the bonded and natural interfaces.

**Table 2:** Summing up the evolution of displacement of each specimen tested. Values are in (mm).

Specimen	*d*_max_ (first cycle)	*d*_max_ (last cycle)	Δ*d*_max%_
AlSi Ln	0.4065	0.4507	+10.9%
AlSi Hn	0.3321	0.3877	+16.7%
AlSi Lb	0.2404	0.2728	+13.5%
AlSi Hb	0.1856	0.2134	+15.0%

**Figure 13:**
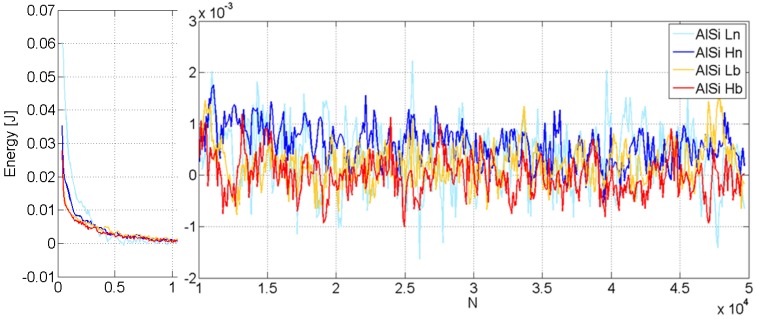
Mechanical work *W*_absorbed(i)_ absorbed in each loading cycle *vs.* the total number *N* of cycles; different y-scales are used for *N* < 10^4^ cycles and for *N* > 10^4^ cycles.

Since the load and displacement were continuously monitored during the tests, the mechanical work and work rate dissipated by the structure at each cycle *i* could be easily calculated. It can be defined as:

(5) $$W_{dissipated\left(i\right)}=\left|W_{load\left(i\right)}\right|-\left|W_{unload\left(i\right)}\right|$$

where *W*_load(i)_ is the work made by the machine during loading and *W*_unload(i)_ is the mechanical work made by the structure during unloading. It is observable that each tube spends the first 10,000 cycles stabilizing its behavior, with a significant dissipation of energy per cycle (Figure 13a). At each cycle, the work is dissipated partly by degeneration phenomena (fatigue) inside the metal foam and the outer structure (plastic work, micro-fractures) and partly by frictional work. The two structures allow frictional work because they are not glued at the interface. When the number of cycles exceeds 20,000 (Figure 13b), the dissipation energy stabilizes to a constant albeit small positive average value for all structures. Again, larger values were recorded for the two structures with no bonding at the interface. This small but constant energy dissipation has very good and interesting effects: it indicates that no catastrophic degeneration phenomena occur in the structure, but no vibration damping properties would be exhibited by the structures if the *W*_dissipated_ average value were equal to zero. 

**Figure 14:**
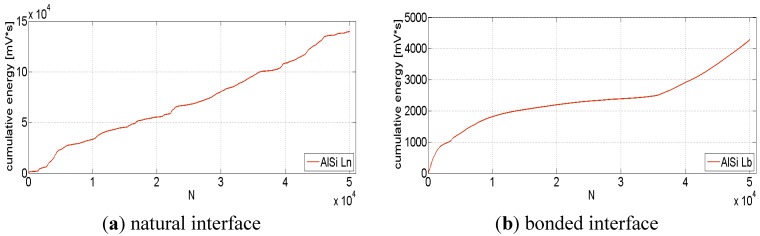
Cumulative energy of the acoustic emission signal during the cyclic test; specimens AlSi Ln (**a**) and AlSi Lb (**b**).

The analysis of the acoustic emissions was also used to further investigate the dissipation phenomena. The attention was focused on the acoustic energy of the signal recorded for each event (hit) acquired during the test. This analysis gives information about any kind of internal dissipation: plastic deformation, crack nucleation and propagation, large fractures and crack jumps, internal friction between foam cell walls and friction between the foam and the tube. For each specimen tested, the evolution of the cumulative energy of the signal (mV·s) were computed during cyclic loading. Figure 14 shows this evolution for the specimens with low foam density, respectively with bonded and free interface. Similar trends are shown for the high-density specimens. According to the literature [23], when a material undergoes fatigue, the AE activity steeply increases in the very first cycles because of adjustment. It then has a slow (almost constant) linear trend during the movement of dislocations and the growth of cracks, and then exponentially increases when a large crack propagates, or the whole section collapses. This behavior was proved for bulk materials (where no frictional work can take place) and there are no previous studies that clearly describe how metal foam-filled structures behave.

Indeed, the AlSi Ln tube exhibits a smooth piecewise linear trend, with some minor fluctuations, since the beginning of the test (Figure 14a). On the contrary, the bonded interface tube (Figure 14b) shows a steepest initial slope and no clear sign that large defects are propagating inside the specimens. The order of magnitude of the noise energy signal for the structure with the free interface is significantly larger. This much larger noise level is entirely due to friction at the interface, which masks and overwhelms any other internal effect. This analysis confirms how the frictional interface can be considered responsible of the high damping and energy absorbing abilities.

## 4. Conclusions

In this paper, the stability of damping and flexural properties of foam-filled tubular structures were, to the authors’ knowledge, studied for the first time. All results indicate a relatively low importance of the foam density and, conversely, a dramatic effect of the tube-foam interface condition.

Cyclic three-point bending tests were performed on foam-filled steel samples with square cross-section (Section 2.2) in order to simulate a number of work cycles and to measure the endurance of these structures. Three closed-cell foam fillings were tested: aluminum AlSi10 foam, hybrid APM foam with low density, hybrid APM foam with high density. The results indicate that there is a lower loading limit, below which no detrimental effects are evident on the endurance of foam-filled structures. If the structures are loaded in cyclic bending below this limit, the behavior of the APM-filled structures seems to be nearly unaffected by the density of the foam. The reason is that the stiffness of the outer tubular case is significantly larger than the foam core, and any change in the core properties is not relevant with respect to the measured bending punch displacement.

When testing the damping properties of the structures before and after the cyclic bending tests (Section 2.3), the results are surprising: the damping ratio DR of all tubes significantly improves, especially at a high-frequency (bending) mode. On the contrary, the dynamic stiffness and the modal mass are virtually unchanged before and after the endurance tests. The increase in damping ratio is due to two mechanisms: hysteresis (*i.e*., damage) of the foam core and frictional work. In the tube filled with AlSi10 foam, where frictional work is present because of the poor interface adhesion between the core and the internal tube surface, the increase in DR after 10,000 bending cycles is very large.

Additional tests were performed in order to further investigate the role of the interface on the stability of the properties of aluminum foam-filled tubes. Round titanium foam-filled tubes were used to compare two different foam densities and two interface conditions: bonded (glued) interface and natural (*i.e.*, completely frictional) interface between tube and core. In free-free modal tests (Section 3.2.2), the glued tubes showed a clear response with predictable natural frequencies. The natural interface tubes had a noisier response, showing many other peaks. The results clearly prove that the foam density has a negligible effect on the damping ratio, whereas the interface condition strongly changes the DR of these structures, with much larger values for tubes with frictional interface.

These round titanium tubes were also tested in cyclic three-point bending with 50,000 cycles (Section 3.3). As already verified for the square tubes, there is a load limit below which the displacement is nearly constant with constant load, and no macroscopic damage is expected. The results show that higher density samples are stiffer (as was obvious) and that structures with a glued interface are stiffer than structures with a frictional interface. In terms of stability of the bending response, *i.e*., moving from the first to the last loading cycle, the stiffness of structures with a lower density is more constant over time, while there is no clear difference between the bonded and natural interfaces. However, the energy dissipated in bending by the structures with frictional interface is larger. This confirms the important role of interface friction in energy-absorption phenomena.
